# Dissecting rifampicin heteroresistance in Mycobacterium tuberculosis: integrating whole-genome sequencing with phenotypic and clonal validation

**DOI:** 10.1099/jmm.0.002048

**Published:** 2025-07-30

**Authors:** Katherine Vallejos-Sanchez, Diego A. Taquiri-Díaz, Omar A. Romero-Rodriguez, A. Paula Vargas-Ruiz, Jorge Coronel, Arturo Torres, Jose L. Perez-Martinez, Adiana Ochoa-Ortiz, Robert H. Gilman, Louis Grandjean, Martin Cohen-Gonsaud, Mirko Zimic, Patricia Sheen

**Affiliations:** 1 Laboratorio de Bioinformática, Biología Molecular y Desarrollos Tecnológicos, Laboratorios de Investigación y Desarrollo, Facultad de Ciencias e Ingeniería, Universidad Peruana Cayetano Heredia, San Martín de Porres 15102, Lima, Peru; 2 Centre de Biologie Structurale, CNRS, INSERM, Montpellier 34090 , France; 3 Department of Infection, Immunity and Inflammation, Institute of Child Health, University College London, London WC1N 1EH, UK; 4 International Health Department, Johns Hopkins Bloomberg School of Public Health, Baltimore, MD, USA

**Keywords:** heteroresistance, mixed populations, rifampicin, tuberculosis, whole-genome sequencing (WGS)

## Abstract

**Introduction.** This study underscores the critical role of identifying heteroresistant infections of *Mycobacterium tuberculosis* (Mtb) in enhancing the diagnostics of tuberculosis (TB). These conditions complicate diagnostics and treatment, underlining the need for advanced techniques to detect and characterize resistant populations effectively.

**Hypothesis/Gap statement.** Current diagnostics may fail to identify heteroresistance and mixed infections, limiting the understanding of their impact on treatment outcomes.

**Aim.** This pilot study aimed to phenotypically and genotypically characterize rifampicin-heteroresistant clinical isolates and assess their genetic diversity and resistance patterns.

**Methodology.** A retrospective analysis of 2,917 Mtb genomes from Peru (1999–2020) was conducted using MTBseq and TB-Profiler. Techniques included indirect microscopic observation drug susceptibility, MIC determination via tetrazolium microplate assay, agar proportion method and sequencing. From each clinical isolate, three colonies were isolated from both rifampicin-supplemented (1 µg mL^−1^) and drug-free media for subsequent phenotypic and genotypic characterization, including *rpoB* sequencing.

**Results.** Of the 2,917 genomes analysed, 14.6% were classified as mixed infections, 3.8% exhibited heteroresistance to at least 1 drug between 21 antibiotics analysed and 0.79% were rifampicin-heteroresistant. Colonies from rifampicin-supplemented media displayed high resistance (MIC >1 µg mL^−1^) with mutations such as S450L in the RpoB protein. In contrast, those from drug-free media exhibited sensitivity to rifampicin (MIC <1 µg ml^−1^), harbouring other RpoB mutations including D435Y, L452P and L430P. Notably, some colonies retained WT RpoB sequences, suggesting a diversity of subpopulations within isolates.

**Conclusion.** Whole-genome sequencing and phenotypic analysis confirmed the coexistence of rifampicin-susceptible and rifampicin-resistant Mtb populations within single clinical isolates. Subculturing in drug-free media favoured the selection of sensitive strains, emphasizing the critical need for advanced diagnostic tools to accurately detect and characterize heteroresistant and mixed infections. These findings pave the way for more targeted treatment strategies to combat antimicrobial resistance in TB.

## Data Summary

Our study used sequencing data from public datasets available in the European Nucleotide Archive under the following project accessions: PRJEB5280, PRJEB32234, PRJEB23245 and PRJEB39837. A full list of raw sequencing data accession numbers is provided in Data S5, available at https://github.com/diego-taquiri/rif-het-tb.

## Introduction

Tuberculosis (TB), caused by *Mycobacterium tuberculosis* (Mtb), remains a leading global health challenge and a significant contributor to antimicrobial resistance [1]. Efforts to control the disease are further complicated by the presence of mixed infections and heteroresistance, phenomena that reflect the pathogen’s genetic diversity and adaptive capabilities [2].

Mixed infections, defined as the simultaneous presence of multiple distinct Mtb strains within a single patient, represent a significant obstacle in TB management. These infections may arise through simultaneous transmission of multiple strains during a single infection event, sequential infections over time or within–host diversification after a single infection [3,5].

Microbiological heterogeneity within a single patient has become an important item to study, due to its relationship with drug tolerance or drug resistance [3]. Mixed infections often lead to heteroresistance – a condition characterized by the coexistence of drug-susceptible and drug-resistant Mtb populations within the same clinical sample [6]. This phenomenon complicates treatment, as resistant strains can thrive and proliferate under selective pressure while susceptible strains are suppressed [7,8]. The global presence of such complex infections represents a critical barrier to eradicating TB [9].

The clinical implications of mixed infections are profound. Patients with multiple Mtb strains are at a higher risk of poor treatment outcomes. Dickman *et al*. [10] reported that patients with multiple Mtb strains showed similar proportions of Mtb smear-positive cultures after 2 and 5 months of treatment. Approximately 22.9% of patients with mixed infections experienced treatment failures after 6 months, and 24% demonstrated heteroresistance, suggesting a strong link between these phenomena and treatment failure [11]. These findings emphasize the need for advanced diagnostic tools capable of detecting mixed infections and profiling their resistance patterns.

Whole-genome sequencing (WGS) has emerged as a transformative tool in TB research, enabling detailed genetic analysis to identify mixed infections and providing comprehensive resistance profiles. Recent studies have revealed that mixed infections, including those resistant to rifampicin (RIF) and isoniazid (INH), constitute ~1% of isolates globally, with rates exceeding 5% in certain countries [12]. This variability underscores the need for region-specific strategies to address the challenges posed by mixed infections.

In Peru, the incidence of multidrug-resistant TB (MDR-TB) increased markedly between 1999 and 2020, rising from 50 to 200–300 cases per 100,000 population [13]. This alarming trend underscores the urgent need for improved diagnostic and treatment strategies. Moreover, Peru’s high MDR/rifampicin-resistant (RR)-TB burden highlights the critical importance of addressing mixed infections and heteroresistance.

This study aims to deepen the understanding of the genotypic and phenotypic diversity in Mtb within individual patients, focusing on primary cultures identified as heteroresistant to RIF based on their WGS and drug susceptibility test (DST). By characterizing these diversities, we can enhance diagnostic accuracy and tailor treatment strategies more effectively, ultimately improving treatment outcomes and reducing the transmission of resistant TB strains.

## Methodology

### *M. tuberculosis* genome data analysis, mixed infections and *in silico* rifampicin heteroresistance determination

WGS data from 2,945 Mtb clinical isolates processed by the Peruvian Tuberculosis group were assembled and analysed using the reference strain H37Rv (NC_000962.3) with the MTBseq [14] and TB-Profiler pipelines (https://tbdr.lshtm.ac.uk/) [15]. These isolates were sequenced as part of previous studies conducted by the Peruvian Tuberculosis Group, and sequencing data have been made publicly available in the European Nucleotide Archive under the project accessions PRJEB5280, PRJEB32234, PRJEB23245 and PRJEB39837. The complete list of individual accession numbers used in this study is provided in Data S5, available in the online Supplementary Material.

The genomes were filtered based on the percentage of mapped reads (using a cutoff of 90%) and an average depth greater than 40X. The genotypic profiles derived from MTBseq and TB-Profiler analyses were compared to gain comprehensive insights into drug resistance. A single-nucleotide polymorphism (SNP) database was also compiled and cross-referenced with the microscopic observation drug susceptibility (MODS) test, which provided phenotypic information from our databases and health centres to determine the RIF susceptibility profile.

For lineage classification, TB-Profiler uses the SNP database consistent with the gold standard regions of the difference classification system proposed by Coll *et al*. [16] and the SNP barcode refined by Napier *et al*. [17], which considers 90 SNPs in the analysis.

Heteroresistance analysis was performed by the TB-Profiler pipeline [15] in its command-line version 4.4.2 with default settings. This software used variant calling on candidate antibiotic resistance genes in TB using its built-in database, TBDB (TB database) [18]. We required a minimum depth of 10 reads for identifying polymorphisms and set the minimum allele frequency for calling a variant at 0.1. Reads cleanup was conducted using Trimmomatic version 0.39, mapping with Burrows-Wheeler Aligner version 0.7.17 and variant calling with FreeBayes version 1.3.5.

### Reactivation of primary cultures of *M. tuberculosis* for drug susceptibility testing

Two groups of four primary cultures, previously characterized by WGS and drug susceptibility profiles determined by MODS, were processed: one classified as rifampicin-heteroresistant and the other classified as rifampicin-susceptible. These isolates were randomly selected.

The rifampicin-heteroresistant group includes different drug susceptibility profiles determined by MODS in the sputum sample: one isolate was reported as susceptible (1R), one as RR-TB (4R) and two as MDR-TB (2 R-3R). The second group included susceptible strains identified through genomic analysis and MODS [codification name CA-1012 (1S), CA0957 (2S), 28832_3#219(3S), 28889_1#17(4S)]. These samples were requested from the Peruvian Tuberculosis group as they are part of the collection for the period 1999–2020.

For the reactivation, an aliquot of the glycerol stock was cultured in 2 mL of Middlebrook 7H9 (DB Difco, USA) liquid medium, supplemented with 10% oleic acid, albumin, dextrose, catalase (OADC) and 0.5% glycerol (7H9-OADC). They were incubated for 7–10 days at 37 °C. Subsequently, 100 µL of this culture was transferred to Middlebrook 7H10 (DB Difco, USA) solid medium, also supplemented with 10% OADC and 0.5% glycerol (7H10-OADC) and then incubated for 21–30 days at 37 °C. These isolates are henceforth referred to as secondary cultures. Mtb H37Rv (pan-susceptible) and DM97 (MDR-TB clinical isolate) were used as control strains.

**Fig. 1.**
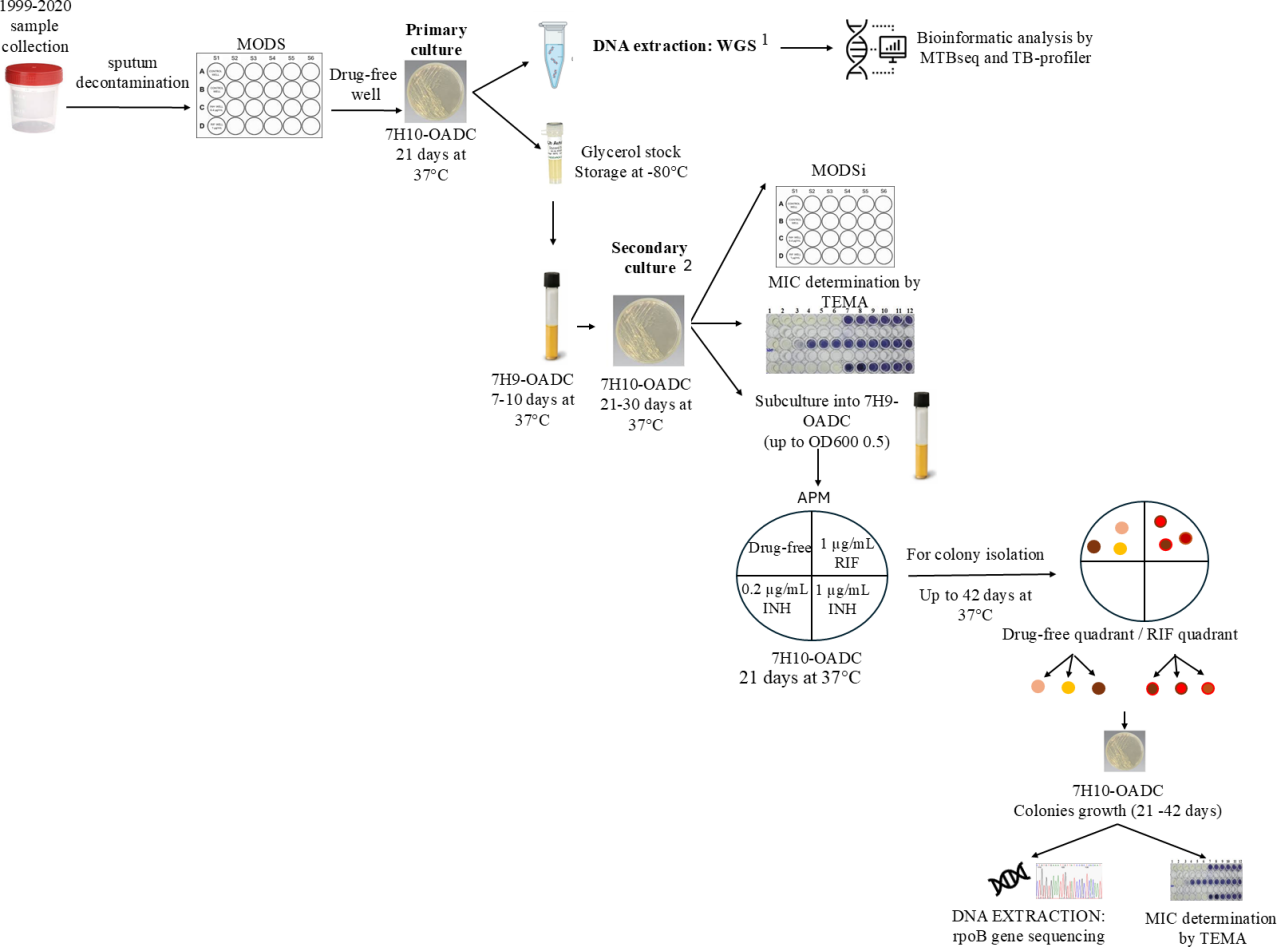
Graphical summary of methodology in the analysis of *M. tuberculosis* heteroresistant/susceptible isolates. ^1^WGS analyzed in this study. ^2^Starting point of cultivations after rifampicin-heteroresistant strains selection.

For each isolate, the susceptibility was determined by indirect microscopic observation drug susceptibility (MODSi), MIC determination by tetrazolium microplate assay (TEMA) and agar proportion method (APM). MODSi offers the earliest visual detection of cord-forming colonies, making it ideal for rapid triage, whereas APM – widely recommended by CLSI (Clinical and Laboratory Standards Institute) [19] – can detect resistant sub-populations as low as ~1% and is, therefore, used as the reference standard for final categorization. In contrast, TEMA, a colorimetric MIC assay, provides quantitative thresholds that are particularly informative in cases with low-level resistance [20]. This integrative use of phenotypic methods enabled robust characterization of the isolates. In cases of discrepancy, results were interpreted using an internal hierarchy based on diagnostic reliability, where APM was prioritized as the reference method, followed by TEMA and the MODSi. This order reflects the broader consensus on the sensitivity and resolution of each method. All procedures were performed at a P3 security level facility. A graphical summary of all procedures is shown in Fig. 1.

### Indirect microscopic observation drug susceptibility

The protocol developed by Caviedes *et al*. [19] was adapted for use with clinical isolates. Briefly, from a 21-day Mtb secondary culture, a suspension equivalent to the McFarland 1 scale was prepared by resuspending approximately two loops of the culture in a mix solution (200 µL Tween 80 10% into 50 mL of sterile distilled water). Then, 5 µL of this suspension was inoculated into a total volume of 5 mL of 7H9-OADC. After that, 900 µL of this dilution were placed into each well of a 24-well plate containing 100 µL of 7H9-OADC, 7H9-OADC with 1 µg mL^−1^ RIF and 7H9-OADC with 0.4 µg mL^−1^ INH. The plates were incubated at 37 °C, with the first examination under an inverted microscope on day 6, followed by monitoring until 21 days.

### MIC determined by TEMA

To determine the MIC for RIF, the TEMA test, which uses 3-(4,5-dimethylthiazol-2-yl)-2,5 diphenyl-tetrazolium bromide, was performed following the previous protocol standardized by our group [20].

Briefly, 96-well plates (Corning REF 3599) were prepared with final antibiotic concentrations as follows: 32 µg mL^−1^ for INH, 16 µg mL^−1^ for RIF, 32 µg mL^−1^ for streptomycin, 128 µg mL^−1^ for ethambutol, 8 µg mL^−1^ for capreomycin and 16 µg mL^−1^ for ciprofloxacin [21] and put them in column 2. Serial dilutions were performed in 100 µL Middlebrook 7H9-OADC from columns 3 to 10. Column 11 was used as a control well without antibiotics.

From a 21- to 30-day Mtb culture, a suspension equivalent to McFarland 1 scale was prepared by resuspending approximately two loops of the culture in a mix solution (200 µL Tween 80 10% into 50 mL of sterile distilled water). A 1 : 25 dilution was made in 7H9-OADC. Then, 100 µL of this dilution was inoculated onto the plate, which was incubated at 37 °C.

On day 5 of incubation, 50 µL of a fresh mixture of 0.1% tetrazolium diluted in absolute ethanol and 10% Tween 80 (1 : 1) was added to the control well and incubated at 37 °C for 24 h. If the well remains yellow, the incubation was extended for another 24 h at 37 °C. If the well remains yellow, the plate is further incubated for up to 20 days. However, if the wells turned purple due to formazan formation, the tetrazolium–Tween 80 was added to all the wells and the colour was assessed after 24 h.

### Determination of the percentage of rifampicin-resistant *M. tuberculosis* population by APM

APM determines the proportion of mutants within a mycobacterial population that are resistant to a specific drug. After 21 days of secondary culture on 7H10-OADC, strains were grown in 7H9-OADC to reach log phase, in the absence of antibiotics. Cultures were then adjusted to a 0.5 McFarland standard using physiological saline solution and serially diluted (10^−2^ and 10^−4^). Approximately 100 µL from each dilution was inoculated in triplicate on 7H10-OADC, across quadrants with varying conditions: (1) drug-free, (2) 1 µg mL^−1^ INH, (3) 0.2 µg mL^−1^ INH and (4) 1 µg mL^−1^ RIF. Cultures were incubated at 37 °C for 3 weeks, and the c.f.u. were counted. Resistance proportion was calculated by comparing colony numbers in antibiotic-supplemented media quadrants to those in the drug-free media quadrant. Clinical isolates were deemed resistant if this percentage was over 1% [19,22], according to the CLSI guidelines. Clinical isolates previously registered as susceptible to INH and RIF were evaluated under the same conditions. Mtb H37Rv (pan-susceptible) and DM97 (MDR-TB clinical isolate) were used as controls.

### Clonal isolation to validate heteroresistance in secondary cultures

**Fig. 2.**
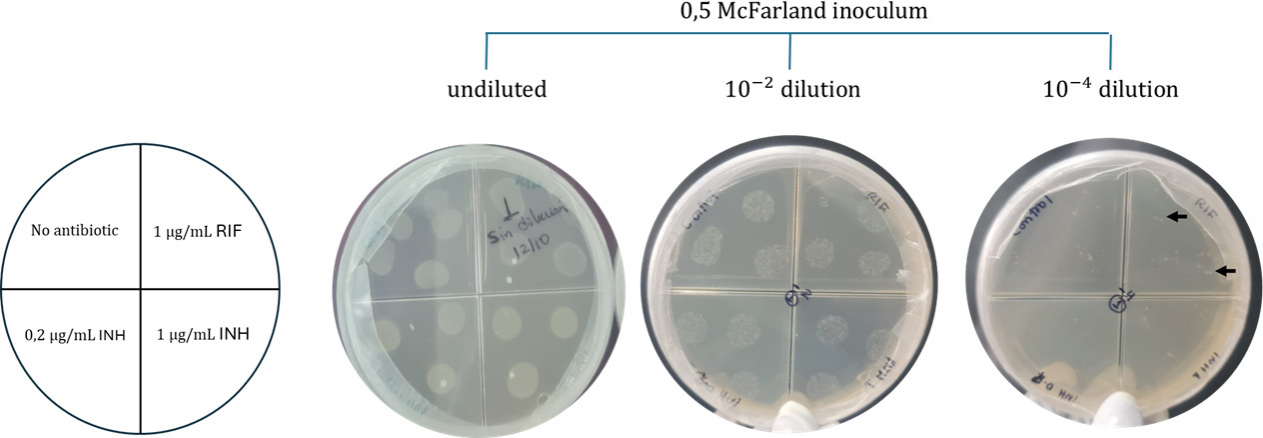
Agar proportion method for *M. tuberculosis* strain. Arrows indicate isolated colonies.

To investigate the presence and selection of pre-existing resistant subpopulations within heteroresistant isolates, six colonies per isolate: three from the drug-free media quadrant and three from the RIF-supplemented media (1 µg mL^−1^) quadrant, using an inoculum corresponding to a 10^−4^ dilution (Fig. 2). They were replicated on 7H10-OADC and incubated for 21–42 days at 37 °C, and a subculture to obtain a full plate was performed under the same conditions. DNA extraction for *rpoB* gene sequencing and MIC determination by TEMA was performed for each colony. This approach enabled us to assess whether the observed growth under selective conditions was associated with specific resistance mutations, thereby validating the WGS-based heteroresistance findings and confirming that the observed variability reflected selection of existing variants rather than *de novo* mutagenesis.

### DNA genomic extraction

Mtb DNA was extracted using the proteinase K digestion method. Briefly, Mtb culture was resuspended in 500 µL TE buffer and inactivated at 100 °C for 30 min. 50 µL of lysozyme (Sigma, USA) (10 mg mL^−1^) was added to the inactivated bacteria and incubated overnight at 37 °C. Subsequently, 75 µL of 10% SDS (J.T.Baker, USA) and 20 µL of proteinase K (Ambion, Life Technologies, USA) (20 mg mL^−1^) were added, followed by incubation at 65 °C for 3 h with homogenization every 20 min for 20 s. After incubation, 100 µL of 5M NaCl (Merck, USA) and 100 µL of pre-heated CTAB/NaCl at 65 °C were added, mixed and incubated at 65 °C for 10 min. Then, 750 µL of phenol–chloroform–isoamyl alcohol (EMD Millipore, USA) (25:24:1) was added, mixed and centrifuged for 5 min at 10,000 r.p.m. The supernatant was recovered into a new tube. To this, 750 µL of chloroform–isoamyl alcohol (24:1) was added, homogenized and centrifuged for 5 min at 10,000 r.p.m., with the supernatant again transferred to a new tube. DNA was precipitated using 1 mL of cold (−20 °C) absolute ethanol (EMD Millipore, USA) and washed with 1 mL of cold (−20 °C) 70% ethanol. The supernatant was discarded, the pellet dried, and DNA resuspended in 50 µL of the elution buffer and then incubated at 55 °C for 15 min to dissolve the pellet. DNA quantification was performed using a spectrophotometer (NanoDrop 2000c).

### *rpoB* gene amplification and sequencing

The PCR mixture included 100 ng of genomic DNA, 2X Phusion Flash High Fidelity PCR Master Mix (Thermo Scientific) and 0.5 µM of each primer F-ext-rpoB (5′-GACAAAATTATCGCGGCGAACG-3′) and R-ext-rpoB (5′TCGCCATAGGACCATTGCCTGA-3′). The cycling conditions were set at 98 °C for 30 s for initial denaturation, followed by 30 cycles of 98 °C for 10 s, 68 °C for 30 s and 72 °C for 2 min (BioRad).

The amplicons were purified with a DNA Clean and Concentrator Kit following the manufacturer’s instructions (Zymo Research). DNA quantification was measured by spectrophotometer (NanoDrop 2000c) and quality by Qubit® dsDNA HS Assay Kit (Thermo Fisher Scientific, Waltham, MA, USA). Then, it was normalized at a final concentration of 50 ng for each sample.

Samples were prepared for sequencing in the Laboratorio de Bioinformática y Biología Molecular - LID-UPCH-Peru, following the manufacturer’s instructions for Oxford Nanopore Technologies. Then, R10.4.1 flow cells were utilized for sequencing on a GridION (ONT, Oxford, UK) for a duration of 72 h. Finally, an analysis for variant calling was performed with the pipeline EPI2ME/wf-amplicon Bioinformatics resources from Oxford Nanopore Technologies Plc (https://github.com/epi2me-labs/wf-amplicon).

## Results

### *M. tuberculosis* genome data analysis, mixed infections and *in silico* rifampicin heteroresistance determination

RIF susceptibility analysis classified 76.35% of MTBseq and 76.24% of TB-Profiler as sensitive strains, indicating similar prediction accuracies by both pipelines. 20.05 and 20.26% strains were classified as genetically and phenotypically RR-TB isolates with MTBseq and TB-Profiler, respectively (agreement percentage 96.4 and 96.5%; coefficient kappa 0.895 and 0.899, respectively) (Table 1).

**Table 1.** Percentage concordance between phenotypic susceptibility determined by MODS and genotypic susceptibility from WGS for rifampicin, analysed by MTBseq and TB-Profiler

*N*=**2,917**	MTBseq		TB-Profiler	
MODS	Resistant (%)	Sensitive (%)	Resistant (%)	Sensitive (%)
Resistant (%)	20.05	1.03	20.26	0.82
Sensitive (%)	2.57	76.35	2.67	76.24
Agreement (%)	96.4		96.5	
Coef. kappa	0.895		0.898	

The discordance rate between genotypic resistance (the presence of SNPs associated with RR-TB) and phenotypic susceptibility (susceptible to RIF in negative MODS) for both pipelines was 2.57–2.67%. Discordance for strains lacking SNPs (related to RIF susceptible) but testing as RIF resistance in MODS ranged between 1.03 and 0.82% (Table 1).

**Fig. 3.**
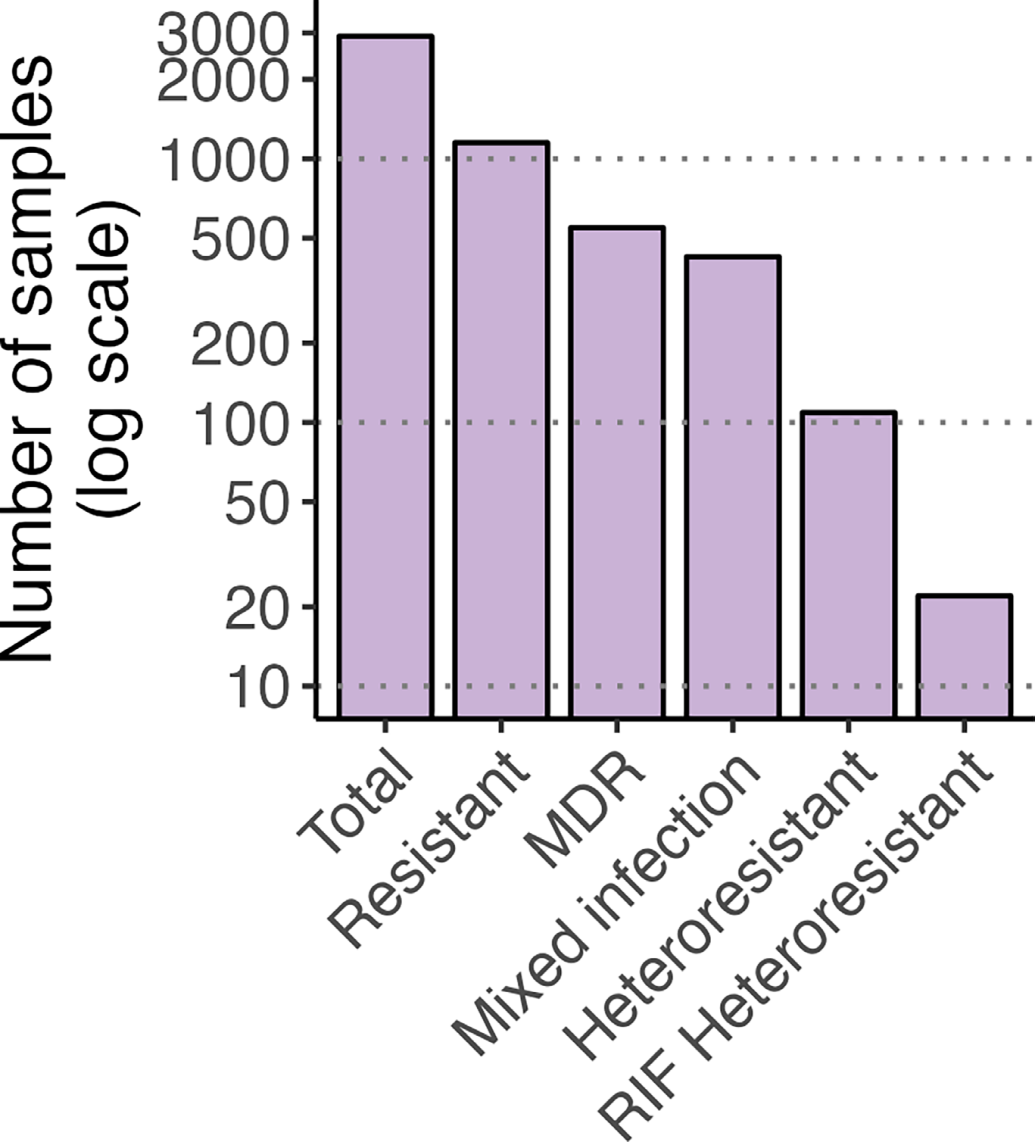
Genotypic resistance profile of 2,917 *M. tuberculosis* clinical isolates from Peru collected between 1999 and 2020. Strains were filtered with over 90% of reads mapped and an average depth exceeding 40X.

2,917 isolates were analysed by TB-Profiler, and the results showed that the frequency of some SNPs in *rpoB* did not represent 100%, so this information opens the possibility that other SNPs are present in lower abundance. Various subpopulations were categorized as follows: isolates resistant to at least one drug (39.5%), MDR-TB (18.8%), mixed infections (14.6%), heteroresistant to at least one drug (3.8%) and specifically rifampicin-heteroresistant (0.79%). Additionally, 9,5% of the isolates were identified as heteroresistant and MDR-TB at the same time (Fig. 3).

Twenty-three isolates were detected as rifampicin-heteroresistant, and it is noteworthy that these isolates have between one to three SNPs in the *rpoB* gene, with SNP frequency ranging from 11 to 85%, as detailed in Table 2. In this table, allelic change are reported as amino acid substitutions to reflect their protein-level consequences. It is also important to note that some isolates contain a single sublineage, while others have up to two mycobacterial sublineages per isolate.

**Table 2.** Genotypic characteristics of clinical isolates characterized as rifampicin-heteroresistant by genomic analysis by TB-Profiler and MTBseq

Clinical isolate ID†	MODS*	MTBseq	TB-Profiler				
		RIF_RpoB (allele proportion)	RIF_RpoB (allele proportion)	Main lineage‡	Sublineage	No. of sublineage	DR type
LI2174109	RR	S450L (0.8)	S450L (0.80)	4	4.3.3	1	MDR
PLE-0891	RR	S450L (1.0)	T400A (0.18), S450L (1.00)	4	4.3.2	1	MDR
PMFR-0719	RR	M434I (0.84), D435G (0.83), P454L (0.88)	M434I (0.84), D435G (0.85)	4	4.9; 4.3.4.1	2	MDR
PMFR-0732	MDR	D435Y (1.0), V695L (0.99)	D435Y (1.00), A451V (0.20)	4	4	1	MDR
**PMFR-0737** **4R**	RR	--	L430P (0.11), H445D (0.64), L452P (0.17)	2	2.2.1	1	RR
PMOP-0526	MDR	S450L (0.85)	S450L (0.85)	4	4.3.3	1	MDR
PMOP-0618	MDR	S450L (1.0)	S450L (1.00), I480V (0.41)	4	4.1.2.1	1	Pre-XDR
PSLM-0811	MDR	S450A (0.75), S450L (1.0), V695L (1.0)	S450V (0.74)	4	4	1	Pre-XDR
PSLM-0843	MDR	S450L (0.78)	S450L (0.79)	4	4.3.3	1	MDR
PTAN-0241	MDR	S450L (1.0)	S450L (1.00), E761D (0.69)	4	4.3.4.1	1	MDR
28832_3#257	MDR	--	S450L (0.71)	4	4.3.3; 4.3.2	2	MDR
**CA-0116** **1R**	Sus	D435Y (0.8)	D435Y(0.79), L452P (0.16)	1	1.2.1.2.1	1	MDR
28832_4#246	Sus	V695L (0.76)	S450L (0.32)	4	4.3.4.2	1	MDR
28832_4#250	MDR	--	S450L (0.70)	4	4.3.4.2	1	MDR
28832_4#318	Sus	D435V (0.84)	D435V (0.82)	4	4.4.1.1; 4.3.3	2	MDR
28889_1#38	MDR	S450L (1.0), I480V (0.8)	S450L (1.00), I480V (0.77), R552C (0.17)	4	4.1.1	1	MDR
28889_1#95	MDR	D435Y (1.0), V695L (1.0)	D435Y (1.00), L452Q (0.25)	4	4	1	MDR
**29544_1#13** **3R**	MDR	--	S450L (0.24)	4	4.3.4.2; 4.1.2.1	2	MDR
**29544_1#232** **2R**	MDR	S450L (0.81)	S450F (0.83)	4	4.1.2.1; 4.1.1	2	Pre-XDR
29544_1#316	Sus	--	D435V (0.42)	4	4.3.3; 4.3.2	2	MDR
29544_1#337	MDR	--	D435V (0.45)	4	4.3.3; 4.3.2	2	MDR
29544_1#6	MDR	--	D435V (0.26)	4	4.3.3; 4.1.2.1	2	MDR
28832_3#91	RR	S441L (0.81)	S441L (0.82), L452P (0.18)	4	4.8	1	RR-TB

*MODS: Susceptibility profile as determined by MODS for the Peruvian tuberculosis group or primary health centres. Sus, susceptible; RR, rifampicin resistant; MDR, multidrug resistant; pre-XDR, pre-extensively drug-resistant.

†Isolates randomly selected for experimental analysis are codified as 1R, 2R, 3R and 4R, and they are shown in bold type.

‡Lineage 1, Indo-Oceanic; lineage 2, East-Asian; lineage 4, Euro-American.

### Indirect microscopic observation drug susceptibility

In the rifampicin-heteroresistant group, resistance to RIF was observed in two out of four samples by day 6, increasing to three out of four by day 14 post-inoculation (Data S2). One of them, isolate 3R, exhibited slower growth compared to other isolates by day 14, while isolate 1R showed growth by day 21 (data not shown).

In the susceptible isolates group, all isolates remained sensitive throughout the same period. Our H37Rv pan-sensitive control strain showed no growth in the rifampicin-supplemented well until day 14, whereas the MDR-TB control strain demonstrated growth by day 6 in the presence of RIF. Results for INH (0.4 µg mL^−1^) are also shown in Data S2; thus, we determined the MDR-TB phenotype for clinical isolates 1R, 2R and 3R, while clinical isolate 4R is RR-TB.

### MIC determined by TEMA

TEMA assay was performed in our study, and strains with a discrepant phenotype in the rifampicin-heteroresistant group were found, as shown in Data S3. In the rifampicin-heteroresistant group, isolate 1R maintained a susceptibility profile, isolate 2R was reported as RR-TB (MIC >16 µg mL^−1^), while isolates 3R and 4R, discrepant to the primary culture, were reported as susceptible (MIC=0.063 µg mL^−1^).

None of the susceptible strains were reported as RR-TB, consistent with their initial profile. Pan-sensitive H37Rv and clinical DM97 MDR-TB strain maintained their profile.

### Determination of the percentage of rifampicin-resistant *M. tuberculosis* population by APM

The genotypically rifampicin-heteroresistant group had a resistant proportion from 2.5 to 85%. All susceptible clinical isolates keep the susceptible profile (Data S4). The pan-sensitive H37Rv strain grew only in the drug-free media quadrant, while the DM97 MDR-TB strain exhibited consistent growth in quadrants supplemented with RIF (1 µg mL^−1^) and INH (0.2 µg mL^−1^ and 1 µg mL^−1^).

### Clonal isolation to validate heteroresistance in secondary cultures

Upon colony isolation, it was observed that 50% exhibited strong phenotype differences between colonies from the drug-free quadrant and those from the rifampicin-supplemented (1 µg mL^−1^) quadrant (Table 3). In the isolates, 3R and 4R, colonies from the drug-free quadrant were susceptible (MIC <1 µg mL^−1^), whereas colonies from the rifampicin-supplemented quadrant showed resistance (MIC >16 µg mL^−1^). Colonies from both quadrants of isolate 2R displayed identical resistance patterns to RIF (MIC >16 µg mL^−1^). In contrast, isolated 1R showed varied RIF susceptibility profiles, but with a slight difference between colonies isolated from the drug-free quadrant (MIC <0.063 µg mL^−1^) and those isolated from the rifampicin-supplemented quadrant (MIC=0.125 µg mL^−1^). Susceptible strains keep the same drug profile including the pan-sensitive H37Rv.

**Table 3.** MIC determined by TEMA in selected *M. tuberculosis* clinical isolates

Strain ID^*^	Mtb clinical isolates†		Colonies					
	DR genotypic status	MIC TEMA RIF (µg mL^−1^)	Colony ID Free-drug quadrant	MIC TEMA RIF (µg mL^−1^)	Phenotypic status	Colony ID RIF quadrant	MIC TEMA RIF (µg mL^−1^)	Phenotypic status
1R	RIF heteroresistant	0.125	1	<0.063	Sus	1	0.125	Sus
			2	<0.063	Sus	2	0.125	Sus
			3	<0.063	Sus	3	0.125	Sus
2R	RIF heteroresistant	>16	1	>16	Res	1	>16	Res
			2	>16	Res	2	>16	Res
			3	>16	Res	3	>16	Res
3R	RIF heteroresistant	0.063	1	0.063	Sus	1	>16	Res
			2	0.063	Sus	2	>16	Res
			3	0.063	Sus	3	>16	Res
4R	RIF heteroresistant	0.063	1	0.125	Sus	1	>16	Res
			2	0.125	Sus	2	>16	Res
			3	0.125	Sus	3	>16	Res
1S	Susceptible	0.063	1	0.125	Sus	–	–	
			2	0.063	Sus	–	–	
			3	0.063	Sus	–	–	
2S	Susceptible	0.063	1	0.125	Sus	–	–	
			2	0.063	Sus	–	–	
			3	0.063	Sus	–	–	
3S	Susceptible	0.25	1	0.125	Sus	–	–	
			2	0.125	Sus	–	–	
			3	0.125	Sus	–	–	
4S	Susceptible	0.063	1	0.063	Sus	–	–	
			2	0.063	Sus	–	–	
			3	0.063	Sus	–	–	
H37Rv	Susceptible	0.063	1	<0.063	Sus	–	–	
			2	<0.063	Sus	–	–	
			3	<0.063	Sus	–	–	

*Isolates 1R, 2R, 3R and 4R were randomly chosen from Table 2, while isolates 1S, 2S, 3S and 4S were selected from those classified as susceptible by TB-Profiler and MODS.

†Among the isolates classified by TB-Profiler as rifampicin-heteroresistant based on genomic analysis, one susceptible isolate, one RR-TB and two MDR-TB isolates – determined by MODS from sputum samples – were randomly selected for evaluation.

Sus, susceptible; Res, resistant.

### DNA extraction, *rpoB* gene amplification and sequencing

DNA concentrations ranged from 50 to 200 ng µL^−1^ with purity ratios of 260/280 and 260/230 both between 1.8 and 2.0. For PCR, concentrations between 10 to 100 ng of DNA per sample were used, amplifying a 3,728 bp product covering a full-length *rpoB* gene. Allelic changes arereported as amino acid substitutions, as previously described. Synonymous RpoB mutations, including A1075A (T3225C), A36A (T108C), D103D (C309T) and P483P (T1449C), are not included in Table 4.

**Table 4.** Genotype and phenotype comparison involved primary culture, secondary culture and colonies from selected clinical isolates

Isolate ID	Analysis from primary culture				RIF susceptibility from secondary culture			Colony isolation from secondary culture					
	MODS RIF	RpoB MTBseq (%)	RpoB TB-Profiler (%)	Genotypic status TB-Profiler	MODS i	TEMA	APM (% resistance)	Drug-free quadrant			RIF quadrant		
								Colony ID	TEMA	RpoB sequence	Colony ID	TEMA	RpoB sequence
1R	Sus	D435Y (80) WT (20)	D435Y (79), L452P (16) WT (5)	RIF heterores	Res	Sus	Res (2.5)	1	Sus	D435Y	1	Sus	D435Y
								2	Sus	WT	2	Sus	D435Y
								3	Sus	WT	3	Sus	D435Y
2R	Res	S450L (81) WT (19)	S450F (83) WT (17)	RIF heterores	Res	Res	Res (85)	1	Res	S450F	1	Res	S450F
								2	Res	S450F	2	Res	S450F
								3	Res	S450F	3	Res	S450F
3R	Res	–	S450L (24) WT(76)	RIF heterores	Res	Sus	Res (1.8)	1	Sus	WT	1	Res	S450L
								2	Sus	WT	2	Res	S450L
								3	Sus	WT	3	Res	S450L
4R	Res	–	L430P (11), H445D (64), L452P (17) WT (8)	RIF heterores	Res	Sus	Res (53)	1	Sus	L452P	1	Res	S450L
								2	Sus	L430P	2	Res	S450L
								3	Sus	L452P	3	Res	S450L
1S	Sus	–	–	Sus	Sus	Sus	Sus	1	Sus	WT	–	–	–
								2	Sus	WT	–	–	–
								3	Sus	WT	–	–	–
2S	Sus	–	–	Sus	Sus	Sus	Sus	1	Sus	WT	–	–	–
								2	Sus	WT	–	–	–
								3	Sus	WT	–	–	–
3S	Sus	–	–	Sus	Sus	Sus	Sus	1	Sus	WT	–	–	–
								2	Sus	WT	–	–	–
								3	Sus	WT	–	–	–
4S	Sus	–	–	Sus	Sus	Sus	Sus	1	Sus	V695L	–	–	–
								2	Sus	V695L	–	–	–
								3	Sus	V695L	–	–	–
H37Rv	Sus	–	–	pan-S	Sus	Sus	Sus	1	Sus	WT	–	–	–
								2	Sus	WT	–	–	–
								3	Sus	WT	–	–	–

L430P, D435Y, H445D and L452P substitutions in RpoB are considered borderline mutations that confer low-level RIF resistance WHO (2023).

Sus, susceptible; Res, resistant; RIF cut off, 1 µg ml−1; Heterores, heteroresistant.

Table 4 summarizes all the analyses performed for this study, considering the initial DST for the primary culture, the genomic analysis based on WGS with two software: MTBseq and TB-Profiler. This table includes the phenotypic analysis for secondary culture after cryopreservation, considering MODSi, DST determined by TEMA and the APM. Finally, a characterization phenotypically for RIF susceptibility by TEMA and *rpoB* sequencing was performed for colonies obtained from the isolates belonging to secondary cultures of Mtb clinical strains.

Isolate 1R carried the borderline RpoB D435Y mutation (80%) together with a minority L452P clone (16%). Despite the mutation, MIC remained ≤0.125 µg mL⁻¹ in TEMA, consistent with incomplete steric hindrance of rifampicin binding. Whole-gene sequencing confirmed the absence of *rpoA*/*rpoC* compensatory mutations. Slow growth (first colonies on MODSi day 21) is compatible with the fitness cost previously described for D435Y lineages.

Isolate 2R analysed by MTBseq and TB-Profiler showed the S450L and S450F RpoB mutations, respectively, both with abundances over 80%. Two sublineages, 4.1.2.1 and 4.1.1, were predicted to be harboured in this isolate. Experimentally, the evaluation of this isolate showed consistency as RR-TB across all tests. Colonies from both quadrants, drug-free and rifampicin-supplemented, maintained the RR-TB profile with the S450F mutation in the RpoB. This mutation appears to be fixed within the population, as only bacilli carrying this mutation could be recovered from the colonies, regardless of drug exposure at the time of isolation.

Isolate 3R revealed the S450L mutation at a frequency of 24% according to TB-Profiler, while no mutations were detected by MTBseq. Two sublineages, 4.3.4.2 and 4.1.2.1, were identified in the sample. TEMA classified this isolate as rifampicin-susceptible; however, colonies from rifampicin-supplemented quadrants harboured the S450L mutation and exhibited an RR-TB phenotype, whereas colonies from the drug-free quadrants displayed RIF susceptibility.

Isolate 4R was identified by TB-Profiler as belonging to lineage 2.2.1 and harbouring the L430P, H445D and L452P mutations, which were not detected by MTBseq. In secondary culture, both the MODSi and APM tests indicated RR-TB, whereas TEMA classified the isolate as rifampicin-susceptible. Colonies from the drug-free quadrant carried the L452P and L430P mutations and were classified as rifampicin-susceptible by TEMA, while colonies from the rifampicin-supplemented quadrant, which harboured the S450L mutation, exhibited resistance to RIF.

Clinical isolates identified as rifampicin-susceptible in primary culture remained susceptible in subsequent DST, with no colonies obtained in the APM. No mutations related to RIF resistance were reported for these strains. The pan-sensitive H37Rv strain maintained a rifampicin-susceptible profile throughout all tests performed, similar to the clinical isolates.

## Discussion

Globally, although the proportion of MDR/RR-TB cases has declined, our findings highlight the persistent and escalating challenge in regions like Peru, where RR-TB cases have increased significantly by 80.7% from 2021 to 2022 according to the latest statistics [23]. Our comprehensive study underscores the significant prevalence and complexity of heteroresistance in TB, particularly in RIF resistance.

For RIF, a potent anti-TB drug, resistance is primarily due to mutations in the *rpoB* gene’s RIF resistance-determining region, with reported frequencies ranging from 85.2 to 90% [24,25]. In our study, this accounted for 96.1% of the RR-TB population. In a related observation, Zheng *et al.* [26] found that 3.8% of RR-TB clinical isolates had no mutations in the *rpoB* gene, closely matching our finding of 3.9%. In the rifampicin-susceptible population, Su *et al.* [24] reported that 9% of TB strains had *rpoB* mutations, compared to 3.4% in our study when analysed with the MODS test. In addition, Aung *et al.* [27] found a *rpoB* mutation rate of 10.1% in clinical isolates prior to RIF treatment, indicating the complexity of the genetic landscape.

This genetic complexity is also evident in mixed infections, which cannot always be detected by traditional clinical diagnostic methodologies. For example, in Peruvian samples, the percentage of mixed infections was 1.4%, determined by spoligotyping and 15-locus MIRU-VNTR analysis, however, 23.5% of the MDR-TB isolates analysed in that study could not be assigned to a lineage [28]. The prevalence of mixed infections varies greatly by geographic location, ranging from as low as 0.4% to as high as 57%, with higher rates generally observed in regions with intensive TB transmission [29,30], being more important in regions where prevalence is high and especially when related to heteroresistance. Sobkowiak *et al.* [31] used WGS to identify mixed infections *in silico* when the minor strain exceeded 10%, finding a frequency of ~10% for this population. Our study used TB-Profiler and MTBseq pipelines, which both differ in sensitivity and intended application. TB-Profiler is optimized for clinical use and prioritizes the detection of resistance-associated mutations, including those at intermediate frequencies or with uncertain significance [18]. By incorporating updated World Health Organization (WHO) catalogues, MTBseq, in contrast, applies stringent filters for variant quality and frequency, favouring conservative SNP calls for robust phylogenomic and transmission analyses [14]. As a result, low-frequency or novel variants present in mixed infections may be filtered out by default in MTBseq, as shown in Table 2 compared with TB-Profiler.

Based on our study objectives, we selected TB-Profiler due to its higher sensitivity for detecting heteroresistant subpopulations. This allowed us to identify mixed infections in 14.6% of the isolates and heteroresistance to at least one of 21 evaluated drugs in 3.8% of the Peruvian clinical isolates. These findings highlight the complexity of TB infections and emphasize the need for advanced bioinformatic tools to uncover such variations. Moreover, 23 isolates (23/2,917=0.79%) were identified as rifampicin-heteroresistant by TB-Profiler; of these, 91.3% (21/23 isolates) belonged to lineage 4 (Euro-American), while 4.35% (1/23) belonged to lineages 1 (Indo-Oceanic) and 2 (East-Asian) each. Among the lineage 4 isolates, 65.2% had a single sublineage, and 34.8% had two sublineages. This is particularly critical given the geographic and lineage-specific variability in drug resistance and transmissibility of Mtb strains, with certain lineages such as lineage 2 and lineage 4 showing higher risks of drug resistance and widespread distribution, respectively [32,35]. Our results are consistent with Hofmann-Thiel S *et al.* [6], who proposed two mechanisms for heteroresistance: superinfection by two different lineages or diversification of a single lineage into susceptible and resistant strains, the latter being more likely to relapse.

Additionally, our results reflect the most prevalent TB lineages circulating in Peru, as reported by Barletta *et al.* [28] and Grandjean *et al.* [36]. Lineage 4 accounts for 58.5–68% of cases, while lineage 2, the second most common, represents 9–16.4% of cases. These lineages also dominate among isolates with extensively drug-resistant profiles [37].

Among the isolates analysed, we have isolate 1R, which belongs to sublineage 1.2.1.2.2.1, which is associated with Southeast Asia [38] and has low prevalence in Peru. It is important to note that in secondary culture for MODSi, this isolate exhibited delayed growth by day 21, unlike other isolates. In the APM, isolate 1R showed a ratio of 2.5%, while isolate 3R, which also had a low proportion (1.8%), grew in MODSi by day 14. This indicates that isolate 1R contains slow-growing strains and possesses *rpoB* mutations categorized as borderline [25], complicating the accurate identification of its resistance profile due to partial resistance to RIF. Mutations in the *rpoB* gene may affect the growth rate and vary the relative fitness [39,40]; these mutations can also be influenced by the genetic background and affect the potential transmission [41]. However, the reason why some strains grow later than others remains unclear.

In the analysis of colonies from isolate 3R, two distinct populations were identified: one with WT RpoB exhibiting a susceptible RIF phenotype and the other with the S450L mutation displaying RR-TB. This mutation was present at a frequency of 24% in the WGS analysis from the primary culture and dropped to 1.8% in secondary culture when quantified by the APM, indicating a significant decrease in the population harbouring the mutation. This phenomenon of predominant populations outgrowing minority ones in culture, potentially masking mixed infections – a phenomenon previously reported by Martin *et al.* [42] in conventional liquid cultures and by Metcalfe *et al.* [43] in subcultures carried out in the absence of drugs. Changes in population ratios are likely influenced by *in vitro* sample processing, where subpopulations with differential growth requirements may be selectively amplified or lost depending on culture conditions [44]. In our study, we minimized this effect by limiting cultures to a single revival from frozen stock and verifying key resistance mutations against the primary-sample WGS data. Notably, the only mutation not detected in the primary sample WGS data was RpoB S450L in strain 4R, which instead harboured other mutations within the *rpoB* gene.

Nevertheless, we acknowledge that even minimal handling can alter population structure and, therefore, interpret any observed MIC shifts with caution.

This concern is consistent with the findings of Metcalfe *et al.* [43], who categorized heteroresistant samples into macroheteroresistance (5–95% of the total population) and microheteroresistance (less than 5% of the total population), highlighting that the diversity of heteroresistant subpopulations decreases with serial cultures, especially when it is less than 1%.

In the case of isolate 4R, the genomic analysis performed by TB-Profiler identified three mutations in the RpoB: L430P, H445D, and L452P, with proportions of 11%, 64% and 17%, respectively. In secondary culture, 53% of the population displayed rifampicin-heteroresistance determined by APM. Subsequent analysis of colonies identified mutations S450L, L452P, L430P and H445D (data not shown). The L452P and L430P mutations, isolated from colonies grown in a drug-free medium, showed a susceptible RIF phenotype, while the other mutations, isolated from a drug-supplemented medium, showed a rifampicin-resistant phenotype. This diversity aligns with characteristics attributed to lineage 2, which is known for higher mutation rates [45,47]. Jamieson *et al.* [48] also found the L430P mutation confers a rifampicin-susceptible phenotype, as do the H445L, H445N and D435G-S441L mutations. Meanwhile, Salaam-Dreyer *et al.* [49] found this mutation, L430P, in 13.9% of RR-TB isolates compared to 1.1% in MDR-TB isolates, indicating that it confers low-level resistance. In the same study, in ten clinical isolates with the L430P mutation, seven were phenotypically susceptible at a critical concentration of 0.5 µg mL^−1^.

Moreover, Hofmann-Thiel *et al.* [6], later corroborated by Nimmo *et al.* [47], evaluated the nucleotide diversity in patients who completed treatment without mixed infections in a longitudinal study. They observed the emergence of heteroresistant populations that either became fixed or persisted over time. In the same study, they noted the presence of two resistant subpopulations whose RpoB frequencies fluctuated during treatment. Significant mutations identified included D435V, S450L, L430P, H445Y, H445D, L452P and H445R, which coexisted for weeks or months within the same patient. Despite this, no association was found between mixed infections and unfavourable outcomes, but opposite findings were found by Shin *et al.* [50] and Cohen *et al.* [51]. The mutations S450L, L430P, H445D and L452P were also identified in our isolates, suggesting their implications in the generation of heteroresistant populations.

Remarkably, the MIC for colonies with the RpoB D435Y mutation ranged from <0.063 to 0.125 µg mL^−1^ in our study, compared to Jeon *et al.* [52], who reported a range of 0.5 to 16 µg mL^−1^. For the L452P mutation, we observed an MIC of 0.125 µg mL^−1^, while Jeon *et al.* found it varied from 1 to 16 µg mL^−1^. These mutations are categorized as borderline by the WHO (2023) [25] due to their phenotypic variability, depending on the MIC test applied.

Additionally, our study identified a nonsynonymous T187P mutation in the RpoA and several mutations in the RpoC protein, including H525Q, G594E, R741S, H767P, I832V, E1033K, P1040A and T1230I (see Data S1 for details). The H525Q and P1040A mutations, previously associated with the transmission of RR-TB isolates, are believed to mitigate minor fitness defects caused by the primary S450L mutation in the RpoB [53,54]. However, the WHO’s 2023 catalogue of ‘mutations in MTBC and their association with drug resistance’ did not find an association with RR-TB for some RpoA and RpoC mutations, including G594E found in this study. Furthermore, the impact of other previously proposed compensatory mutations in the *rpoA* or *rpoC* genes remains to be clarified [53,55, 56], leaving their effects on RIF resistance and their role in transmission uncertain.

Ultimately, the persistence and tolerance of bacterial populations, as shown in our study, suggest that some mutations may confer a survival advantage without altering MICs, complicating the eradication of these populations [57], which emphasizes the importance of considering growth rate differences between strains and proportions, which may contribute to the establishment of drug-tolerant populations [3,58,62]. These observations underline the critical importance of early diagnosis to prevent the development of drug-resistant strains [63,64]. Genotyping in clinical samples is recommended to reveal the clonal complexity of Mtb infection, as was performed by Aung *et al.* [27], who detected 5% heteroresistance to RIF in sputum using droplet digital PCR. The proportion of mutant strains showing *rpoB* gene heteroresistance ranged from 20 to 80%, while nearly all strains in RR-TB populations were 85–100% mutant and almost completely resistant to RIF.

Our analysis confirms the complex interplay between genetic mutations and phenotypic expression. It underscores the necessity for enhanced diagnostic techniques that can accurately detect and characterize heteroresistant and mixed infections. Future research should focus on integrating detailed genomic data with clinical outcomes to develop targeted treatment strategies that address both dominant and minor resistant populations, thereby improving patient outcomes and contributing to the global effort to control and eliminate TB.

## Conclusion

WGS revealed the presence of multiple Mtb strains in the sputum samples, which were further validated through colony isolation. During the isolation process, we identified colonies harbouring mutations in the *rpoB* gene with high levels of rifampicin resistance. These colonies were isolated from RIF-supplemented media, highlighting that subculturing in drug-free media may preferentially select for drug-sensitive strains over resistant ones.

These findings emphasize the critical need for advanced techniques capable of accurately detecting and characterizing heteroresistant and mixed infections. Such tools are essential to improve treatment outcomes and combat the challenges posed by antimicrobial resistance in TB.

## Supplementary material

10.1099/jmm.0.002048 Uncited Supplementary Material 1.

## Abbreviations

APM: agar proportion method
CLSI: Clinical and Labratory Standards Institute
DST: drug susceptibility test
INH: isoniazid
MDR-TB: multidrug-resistant TB
MODS: microscopic observation drug susceptibility
MODSi: indirect microscopic-observation drug-susceptibility
Mtb: *Mycobacterium tuberculosis*
OADC: oleic acid, albumin, dextrose, catalase
RIF: rifampicin
*rpoB*: rpoB gene
RpoB: RpoB protein
RR: rifampicin-resistant
SNP: single-nucleotide polymorphism
TB: tuberculosis
TBDB: TB database
TEMA: tetrazolium microplate assay
WHO: World Health Organization
