# Supplementary material for: Dissecting rifampicin heteroresistance in Mycobacterium tuberculosis: integrating whole-genome sequencing with phenotypic and clonal validation
Source: J Med Microbiol. 2025 Jul 30;74(7):002048. doi: 10.1099/jmm.0.002048 (PMC12310241; doi:10.1099/jmm.0.002048)
Supplement: Uncited Supplementary Material 1. [file jmm-74-02048-s001.pdf]

	Genes related to drug resistance																				
**StrainID**	**RIF**	**INH**	**PZA**	**EMB**	**STM**	**FQ**	**MXF**	**OFX**	**LVX**	**CIP**	**AG**	**AMK**	**KAN**	**CAP**	**ETH**	**PAS**	**CS**	**LZD**	**BDQ**	**CFZ**	**DLM**
**LI2174109**	rpoC_c.1626C>G (1.00), rpoC_p.Pro1040Ala (0.79)	fabG1_c.-15 C>T (0.73)	pncA_p. His51Arg (0.86)	-	gid_c. 319dupG (0.19)	-	-	-	-	-	-	-	-	-	fabG1_c.-15 C>T (0.73)	-	-	-	-	-	-
**PLE-0891**	rpoA_p.Thr187Pro (0.19), rpoC_c.1626C>G (1.00), rpoC_p.Ile832Val (0.48)	katG_p. Ala109Val (1.00)	-	-	-	-	-	-	-	-	-	-	-	-	-	-	-	-	-	-	-
**PMFR-0719**	rpoC_c.1626C>G (0.94)	fabG1_c.-15 C>T (0.89)	-	-	-	-	-	-	-	-	-	-	-	-	fabG1_c.-15 C>T (0.89)	-	-	-	-	-	-
**PMFR-0732**	rpoA_c.-88C>T (0.27), rpoA_c.-128C>T (0.22)	katG_p. Ser315Thr (1.00)	-	embB_p. Gly406Ser (1.00)	-	-	-	-	-	-	rrs_n. 1401A>G (1.00)	rrs_n. 1401A>G (1.00)	rrs_n. 1401A>G (1.00)	rrs_n. 1401A>G (1.00)	-	-	-	-	mmpR5_c. 198dupG (1.00)	mmpR5_c. 198dupG (1.00)	-
**PMFR-0737**	rpoC_c.-339T>C (0.99)	-	-	-	rpsL_p. Lys43Arg (1.00)	-	-	-	-	-	-	-	-	-	-	-	-	-	-	-	-
**PMOP-0526**	rpoC_c.1626C>G (1.00), rpoC_p.Pro1040Ala (0.75)	fabG1_c.-15 C>T (0.73)	pncA_p. His51Arg (0.82)	embB_p. Met306Val (0.83)	-	-	-	-	-	-	-	-	-	-	fabG1_c.-15 C>T (0.73)	-	-	-	-	-	-
**PMOP-0618**	rpoC_p.His525Gln (0.38), rpoC_p.Gly594Glu (1.00)	katG_p. Ser315Thr (1.00)	pncA_p. His57Arg (1.00)	embB_p. Met306Ile (1.00)	rpsL_p. Lys43Arg (1.00)	gyrA_p. Asp94Gly (1.00)	gyrA_p. Asp94Gly (1.00)	gyrA_p. Asp94Gly (1.00)	gyrA_p. Asp94Gly (1.00)	gyrA_p. Asp94Gly (1.00)	rrs_n. 1401A>G (1.00)	rrs_n. 1401A>G (1.00)	rrs_n. 1401A>G (1.00)	rrs_n. 1401A>G (1.00)	-	-	ald_c. 464delG (0.65)	-	-	-	-
**PSLM-0811**	rpoA_c.-88C>T (0.25), rpoA_c.-128C>T (0.27)	fabG1_c.-17 G>T (1.00), katG_p. Ser315Asn (0.99)	pncA_p. Ser104Arg (1.00)	embB_p. Met306Ile (1.00)	-	gyrA_p. Asp94Gly (1.00)	gyrA_p. Asp94Gly (1.00)	gyrA_p. Asp94Gly (1.00)	gyrA_p. Asp94Gly (1.00)	gyrA_p. Asp94Gly (1.00)	rrs_n. 1401A>G (1.00)	rrs_n. 1401A>G (1.00)	rrs_n. 1401A>G (1.00)	rrs_n. 1401A>G (1.00)	fabG1_c.-17 G>T (1.00)	-	-	-	-	-	-
**PSLM-0843**	rpoC_c.1626C>G (1.00), rpoC_p.Glu1033Lys (0.25)	fabG1_c.-15 C>T (1.00), inhA_p. Ile21Val (1.00)	pncA_p. His51Arg (1.00)	embB_p. Gly406Ala (1.00)	-	-	-	-	-	-	-	-	eis_c.-10 G>A (1.00)	-	fabG1_c.-15 C>T (1.00)	-	-	-	-	-	-
**PTAN-0241**	rpoC_c.1626C>G (1.00), rpoC_p.Arg741Ser (1.00)	katG_p. Ser315Thr (1.00)	-	-	gid_c. 180delA (1.00)	-	-	-	-	-	-	-	-	-	-	-	-	-	-	-	-
**28832_3#257**	rpoC_c.1626C>G (1.00), rpoC_p.Pro1040Ala (0.64)	fabG1_c.-15 C>T (0.63)	pncA_p. His51Arg (0.73)	-	-	-	-	-	-	-	-	-	-	-	fabG1_c.-15 C>T (0.63)	-	-	-	-	-	-
**28832_3#91**	-	-	-	-	-	-	-	-	-	-	-	-	-	-	-	-	-	-	-	-	-
**28832_4#246**	rpoA_c.-74_-73insCAACCCA (0.25), rpoA_c.-88C>T (0.28), rpoA_c.-128C>T (0.19), rpoC_c.1626C>G (0.19)	katG_p. Ser315Thr (0.23)	pncA_p. Leu116Pro (0.25)	embB_p. Met306Ile (0.34)	-	-	-	-	-	-	-	-	-	-	-	-	-	-	-	-	-
**28832_4#250**	rpoC_c.1626C>G (0.82)	katG_p. Ser315Thr (0.70)	pncA_p. Leu116Pro (0.62)	embB_p. Met306Ile (0.80)	-	-	-	-	-	-	-	-	-	-	-	-	-	-	-	-	-
**28832_4#318**	rpoC_c.1626C>G (0.84)	katG_p. Ser315Thr (0.88)	pncA_p. Gln10Arg (0.78)	embB_p. Tyr319Ser (0.88)	gid_p. Pro84Leu (0.86)	-	-	-	-	-	-	-	-	tlyA_p. Gly232Asp (0.77)	-	-	-	-	-	-	-
**28889_1#38**	rpoC_p.Gly594Glu (1.00), rpoC_p.Thr1230Ile (1.00)	katG_p. Ser315Thr (1.00)	pncA_p. Gln10Pro (1.00)	embB_p. Asp354Ala (1.00)	-	-	-	-	-	-	-	-	-	-	-	-	-	-	-	-	-
**28889_1#95**	rpoA_c.-88C>T (0.57)	katG_p. Ser315Thr (1.00)	-	embB_p. Gly406Ser (1.00)	-	-	-	-	-	-	-	-	-	-	-	-	-	-	mmpR5_c. 198dupG (1.00)	mmpR5_c. 198dupG (1.00)	-
**29544_1#13**	rpoC_p.Gly594Glu (0.92)	katG_p. Ser315Thr (0.19)	-	embB_p. Met306Ile (0.16)	-	-	-	-	-	-	-	-	-	-	-	-	-	-	-	-	-
**29544_1#232**	rpoC_p.Gly594Glu (1.00), rpoC_p.His767Pro (0.76)	fabG1_c.-15 C>T (0.77), katG_p. Ser315Asn (0.79)	pncA_p. Leu4Trp (0.70)	embB_p. Gln497Lys (0.79)	gid_p. Tyr195* (0.79)	gyrA_p. Ser91Pro (0.77)	gyrA_p. Ser91Pro (0.77)	gyrA_p. Ser91Pro (0.77)	gyrA_p. Ser91Pro (0.77)	gyrA_p. Ser91Pro (0.77)	-	-	-	-	fabG1_c.-15 C>T (0.77)	-	-	-	-	-	-
**29544_1#316**	rpoC_c.1626C>G (1.00)	katG_p. Ser315Thr (0.26)	pncA_p. Tyr103* (0.16)	embB_p. Asp354Ala (0.38)	-	-	-	-	-	-	-	-	-	-	-	-	-	-	-	-	-
**29544_1#337**	rpoC_c.1626C>G (1.00)	katG_p. Ser315Thr (0.42)	pncA_p. Tyr103* (0.35)	embB_p. Asp354Ala (0.52)	-	-	-	-	-	-	-	-	-	-	-	-	-	-	-	-	-
**29544_1#6**	rpoC_c.1626C>G (0.41), rpoC_p.Gly594Glu (0.39)	katG_p. Ser315Thr (0.50)	pncA_p. Gln10Arg (0.46)	embB_p. Tyr319Ser (0.50)	gid_p. Pro84Leu (0.56)	-	-	-	-	-	-	-	-	-	-	-	-	-	-	-	-
**CA-0116**	rpoC_c.-339T>C (1.00), rpoC_c.162G>C (1.00), rpoC_p.Ala172Val (1.00), rpoC_c.517C>A (1.00)	katG_p. Ser315Thr (1.00)	-	-	gid_p. Leu79* (1.00)	-	-	-	-	-	-	-	-	-	-	-	-	-	-	-	-

*Abbr. RIF-Rifampicin, INH-Isoniazid, PZA-Pyrazinamide, EMB-Ethambutol, STM-Streptomycin, FQ-Fluoroquinolones, MXF-Moxifloxacin, OFX-Ofloxacin, LVX-Levofloxacin, CIP-Ciprofloxacin, AG-Aminoglycosides, AMK-Amikacin, KAN-Kanamycin, CAP-Capreomycin, ETH-Ethionamide, PAS-Para-aminosalicylic acid, CS-Cycloserine, LZD-Linezolid, BDQ-Bedaquiline, CFZ-Clofazimine, DLM-Delamanid.

Supplementary data 1. Analysis of mutations in drug resistance-related genes in rifampicin-heteroresistant primary *Mycobacterium tuberculosis* isolates using TBprofiler.

Supplementary data 1.xlxs

*Abbr. RIF-Rifampicin, INH-Isoniazid, PZA-Pyrazinamide, EMB-Ethambutol, STM-Streptomycin, FQ-Fluoroquinolones, MXF-Moxifloxacin, OFX-Ofloxacin, LVX-Levofloxacin, CIP-Ciprofloxacin, AG-Aminoglycosides, AMK-Amikacin, KAN-Kanamycin, CAP-Capreomycin, ETH-Ethionamide, PAS-Para-aminosalicylic acid, CS-Cycloserine, LZD-Linezolid, BDQ-Bedaquiline, CFZ-Clofazimine, DLM-Delamanid.

Supplementary data 2. MODS indirect test for rifampicin-heteroresistant and susceptible clinical isolates selected for this study.

STRAIN ID	Genotypic status	MODS indirect*					
		Day 6			Day 14		
		Control No drug	INH (0,4 µg/mL)	RIF (1 µg/mL)	Control No drug	INH (0,4 µg/mL)	RIF (1 µg/mL)
1R	RIF heteroresistant	1	1	0	1	1	0**
2R	RIF heteroresistant	1	1	1	1	1	1
3R	RIF heteroresistant	1	0	0	1	1	1
4R	RIF heteroresistant	1	0	1	1	0	1
1S	susceptible	1	0	0	1	0	0
2S	susceptible	1	0	0	1	0	0
3S	susceptible	1	0	0	1	0	0
4S	susceptible	1	0	0	1	0	0
H37Rv	pan-sensitive	1	0	0	1	0	0
DM97	MDR	1	1	1	1	1	1

*Drugs susceptibility profile by MODS indirect 0= no growth; 1= growth.

**This isolate was susceptible by MODS from the sputum sample, but MDR by MTBseq and TBprofiler analysis. Additionally, this isolate grew on day 21, showing slow growth compared to the other isolates.

Abbr. RIF=Rifampicin, INH=Isoniazid

Supplementary data 3. Minimum inhibitory concentration determined by TEMA in Mtb clinical strains with rifampicin-heteroresistant and susceptible profiles selected for this study.

Strain ID	Genotypic status	INH (µg/mL)	RIF (µg/mL)	STM(µg/mL)	EMB(µg/mL)	CAP(µg/mL)	CIP(µg/mL)
1R	RIF heteroresistant	1	0.125	0.5	2	2	1
2R	RIF heteroresistant	>32	>16	0.25	16	2	0.25
3R	RIF heteroresistant	0.125	0.063	0.125	4	2	0.25
4R	RIF heteroresistant	0.125	0.063	>32	4	2	0.125
1S	susceptible	0.125	0.063	0.125	4	1	0.25
2S	susceptible	0.125	0.063	0.25	4	4	0.25
3S	susceptible	0.125	0.25	0.125	2	4	8
4S	susceptible	0.25	0.063	0,5	8	4	0.25
H37Rv	pan-sensitive	0.125	0.063	0.5	1	1	0.25
DM97	MDR	4	>16	2	8	1	0.25

Abbr. MDR=multidrug resistant, RIF=Rifampicin, INH=Isoniazid, STM=Streptomycin, EMB=Ethambutol, CAP=Capreomycin, CIP=Ciprofloxacin.

Supplementary data 4. Agar-plate proportions method in selected Mtb clinical strains reactivated with rifampicin-heteroresistant and susceptible profiles selected for this study.

STRAIN ID	DR Genotypic status	APM (>0.01=R)*		
		**RIF (1 μg/mL)**	**INH (1 μg/mL)**	**INH (0.2 μg/mL)**
1R	RIF heteroresistant	Res (0.025)	Res (0.56)	Res (1.0)
2R	RIF heteroresistant	Res (0.85)	Res (0.8)	Res (0.91)
3R	RIF heteroresistant	Res (0.018)	Sus	Sus
4R	RIF heteroresistant	Res (0.53)	Sus	Sus
1S	susceptible	Sus	Sus	Sus
2S	susceptible	Sus	Sus	Sus
3S	susceptible	Sus	Sus	Sus
4S	susceptible	Sus	Sus	Res(0.2)
H37Rv	pan-sensitive	Sus	Sus	Sus
DM97	MDR	Res (1.0)	Res (1.0)	Res (1.0)

(*) The proportion of resistance is described for the resistant strains.
Abbr. Res = resistant, Sus = susceptible, DR= Drug-resistant, RIF=Rifampicin, INH=Isoniazid.

Supplementary data 5: Accession numbers for whole genome sequencing (WGS) datasets of clinical Mtb isolates analyzed in this study. Available at: https://github.com/diego-taquiri/rif-het-tb
